# Extraction replica method from thinned transmission electron microscopy samples without floating off carbon films: A case study on reduced activation steel

**DOI:** 10.1111/jmi.70113

**Published:** 2026-05-19

**Authors:** Jack Haley, Alex Dickinson‐Lomas, Qianyi Sun, David Bowden

**Affiliations:** ^1^ Materials Division UK Atomic Energy Authority Oxfordshire UK; ^2^ Materials Department University of Oxford Oxford UK

**Keywords:** extraction replica, ferritic‐martensitic, precipitates, RAFM, steel, TEM

## Abstract

A recipe for producing carbon extraction replicas from twin‐jet electropolished disks is described. This technique allows tracking the analysed region with respect to the bulk sample. An example use of the method as applied to a reduced activation ferritic martensitic (RAFM) steel, ‘UK‐RAFM’, is reported. The RAFM steel described originates from a ∼5 tonne heat produced via electric‐arc furnace (EAF) and was intended to match Eurofer‐97 in chemistry. Here, a detailed analysis of the precipitates within the UK‐RAFM is provided, with particular emphasis to show the variability in chemistry of second‐phase precipitates (SPPs). Comparisons to literature reported compositions of Eurofer‐97 SPPs indicate they are in reasonable agreement; comparisons are also made against thermodynamic models.

## LAY DESCRIPTION

Scientists frequently need to examine very small features inside the structure of metals—the microstructure—to gain insight into how they derive their strength and other mechanical (or chemical) properties. A powerful tool for this is transmission electron microscopy (TEM), which can reveal details over a wide range of scales, typically from 10s of microns to the atomic scale. To prepare samples for TEM, researchers sometimes use a method called “extraction replicas.” These are very thin films that capture the small particles inside metals that are a critical part of their microstructure. Producing samples this way allows these particles to be studied in detail.

Traditionally, making these replicas involves floating carbon films off a surface and transferring them onto a separate grid that can be placed in the microscope. This step leads to ambiguity over where on the sample surface the film originated. In our work, we describe a new way to make extraction replicas directly from specially thinned samples, without needing to float off any carbon films. This makes the process easier to trace the exact location of the replica with respect to the bulk material.

We tested this method on a steel designed for use in nuclear fusion reactors. Fusion reactors require materials that can withstand extreme heat and radiation, and steels known as “reduced activation ferritic martensitic” (RAFM) steels are promising candidates. Our study focused on the tiny particles, called precipitates, that form inside the steel and strongly influence its performance. Using our improved replica technique, we were able to examine these precipitates in detail and compare their composition to both published data and thermodynamic model predictions.

Our findings show that the new method works well and produces results consistent with existing knowledge. Because extraction replicas are widely used in microscopy, this approach could benefit many researchers studying metals and other materials. By making sample preparation easier and more dependable, we hope this technique will help scientists gain clearer insights into the microscopic structures that control how materials behave.

## 1 INTRODUCTION

The mechanical properties of metals can be substantially altered and controlled by the introduction of nanoscale second‐phase precipitates (SPPs) to the matrix.^1^ SPPs can help to stabilise the microstructure by pinning grain boundaries and dislocations, thereby giving alloys enhanced strength. Particularly for structural alloys such as tempered martensitic steels, carbides, nitrides, carbo‐nitrides, intermetallics and oxides are critical for stabilising the microstructure, and for controlling strength and toughness. Therefore, the study of SPPs forms a critical part of the microstructural characterisation of such alloys. Of particular interest is how SPPs are affected by alloy composition, and heat treatment, as well as their stability during exposure to service‐like conditions, including stress, heating, creep, creep fatigue, and corrosion. Crystal structure, chemistry, size, morphology, and number‐density are therefore important characteristics of SPP distributions.

Transmission electron microscopy (TEM) is heavily utilised for studying SPPs. Thin foils, prepared via twin‐jet electropolishing or focussed ion beam (FIB), are routinely studied to image precipitates, dislocations and grain boundaries.^2^, ^3^ One complexity of imaging SPPs in such samples is that they will be embedded within a matrix, thus making robust characterisation of their composition and crystallography difficult. This can be even more challenging when SPPs reside among other defects that exhibit strong contrast in the TEM, such as dislocation‐dense grains or grain boundaries. Although tilting the sample can be performed to suppress such obscuring contrast, this can quickly become tedious if examining a sample with a fine and complex grain morphology. Some SPPs also have characteristics that complicate their analysis. For example, vanadium (carbo)nitrides have a similar density to iron, and although are semi‐coherent (obeying the Baker‐Nutting relationship), have interfaces with the matrix with very little coherency strain.^4^ The consequence of this is that small vanadium carbonitrides will often show very little contrast, if any, in bright field TEM, bright‐/dark‐field two‐beam conditions, or via Z‐contrast. To image such nanoprecipitates, dark‐field imaging in TEM is required, with a small objective aperture to precisely select the appropriate reflection for imaging, and confidently exclude matrix or surface‐oxide reflections, which can otherwise overwhelm the weak‐contrast of very small precipitates.^4^ Scanning transmission electron microscopy (STEM), in conjunction with electron dispersive X‐ray spectroscopy (EDS), electron energy loss spectroscopy (EELS), or diffraction imaging can also reveal such precipitates, but require more lengthy scans to confidently identify small phases dispersed across large areas.

Extraction replicas are an alternative TEM sample type that have been used to study SPPs in metals for decades.^5^, ^6^, ^7^ This method typically comprises deposition of a carbon film ∼100 nm thick on a chemically etched sample (though alternatives to carbon are sometimes used, including amorphous alumina^8^ and polymers^9^). The extraction film of carbon encases precipitates protruding from the sample surface. By chemically or electrolytically removing the material under the carbon, the precipitates and their location remains preserved within the carbon film. The film is collected onto a 3 mm mesh grid, which can then be examined via TEM or SEM. This enables the precipitates to be studied without the complexities of the metal matrix.

To extract the replica, the typical technique is to score a grid pattern into the deposited carbon film using a blade or sharp tweezers, before chemically etching the sample for a second time.^10^ Etchant is then able to penetrate beneath the carbon film via the scored sections, and given enough time, will cause the carbon film to detach and float to the surface. A disadvantage is that the specific location on the substrate that the film originates from is lost. An alternative approach was described by Bergqvist,^6^, ^11^ who carbon coated etched 3 mm disks, before back‐electropolishing the disks until perforation.

This work reports on an alternative approach to producing carbon extraction replica samples directly from thinned twin‐jet electropolished 3 mm disks. This technique has similarities to the process described by Bergqvist,^11^ but produces the replica directly from the already thinned disk. Application of this approach is shown for a reduced activation ferritic martensitic (RAFM) steel.

## 2 METHODS

### 2.1 Carbon extraction replica

The new method is described in the following steps:

1. Prepare a 3 mm disk suitable for twin‐jet electropolishing
2. Identify appropriate conditions for electropolishing
3. Reduce the voltage by ∼10 V from the ‘polishing’ condition, to electropolish in a slightly ‘etching’ condition. This will vary depending on the sample. Alternatively, the etch can be done later, by lightly etching chemically after the twin‐jet polishing (step 4) and before carbon coating (step 5).
4. Twin‐jet electropolish the sample until a small hole perforates
5. Carbon coat one side of the disk to a thickness of ∼100 nm
6. Apply a protective varnish to the carbon‐side of the disk, for example, using Lacomit (Agar‐Scientific). The varnish is easier to apply by reducing its viscosity by mixing in some of the Lacomit remover solution. This application of varnish helps protect the carbon‐layer during the final step.
7. Etch the disk in a suitable etchant, or via electropolishing, to remove material around the hole.
8. Clean the sample of varnish

**FIGURE 1:**
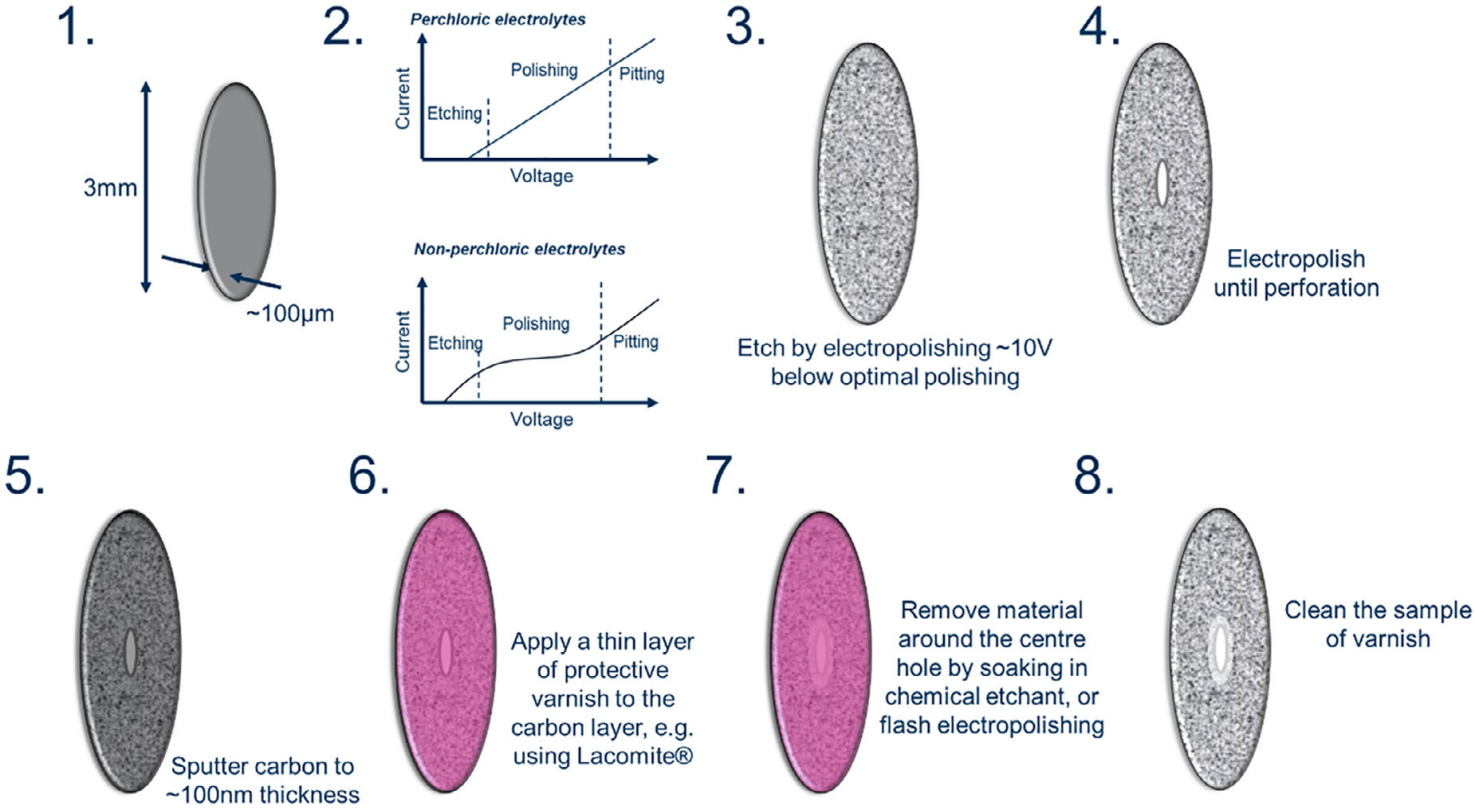
Schematic of the new carbon‐extraction replica technique.

These steps are depicted schematically in Figure 1. Schematics of the conventional method and of the similar Bergqvist method can be found in the review by Rolinska et al.^6^

This method removes step of floating off a carbon film and thus the replica stays attached to the bulk sample. Therefore, the location of the extracted precipitates with respect to the bulk sample can be traced. This means analysis can be conducted on the bulk material before the carbon deposition, for example, TEM imaging or scanning electron microscopy (SEM)‐based analysis such as electron back scatter diffraction (EBSD), and then correlated with the analysis of the precipitates on the carbon film. Another advantage is that by removing material around the hole in the centre of the twin‐jet electropolished disk, some electron‐transparent matrix material is retained. This can therefore also be imaged later, and compared with extraction replica in the same TEM sample and in adjacent locations.

The method is similar to that described by Bergqvist^11^ but is applied to already thinned 3 mm TEM disks. In the steps described, we propose that the electropolishing step be used as the etching step, unlike Bergqvist's method, by changing the electropolishing conditions. Alternatively, a light etch on an already thinned disk can also work, which presents a third advantage in that the replication can be done after analysing a conventionally electropolished foil.

**FIGURE 2:**
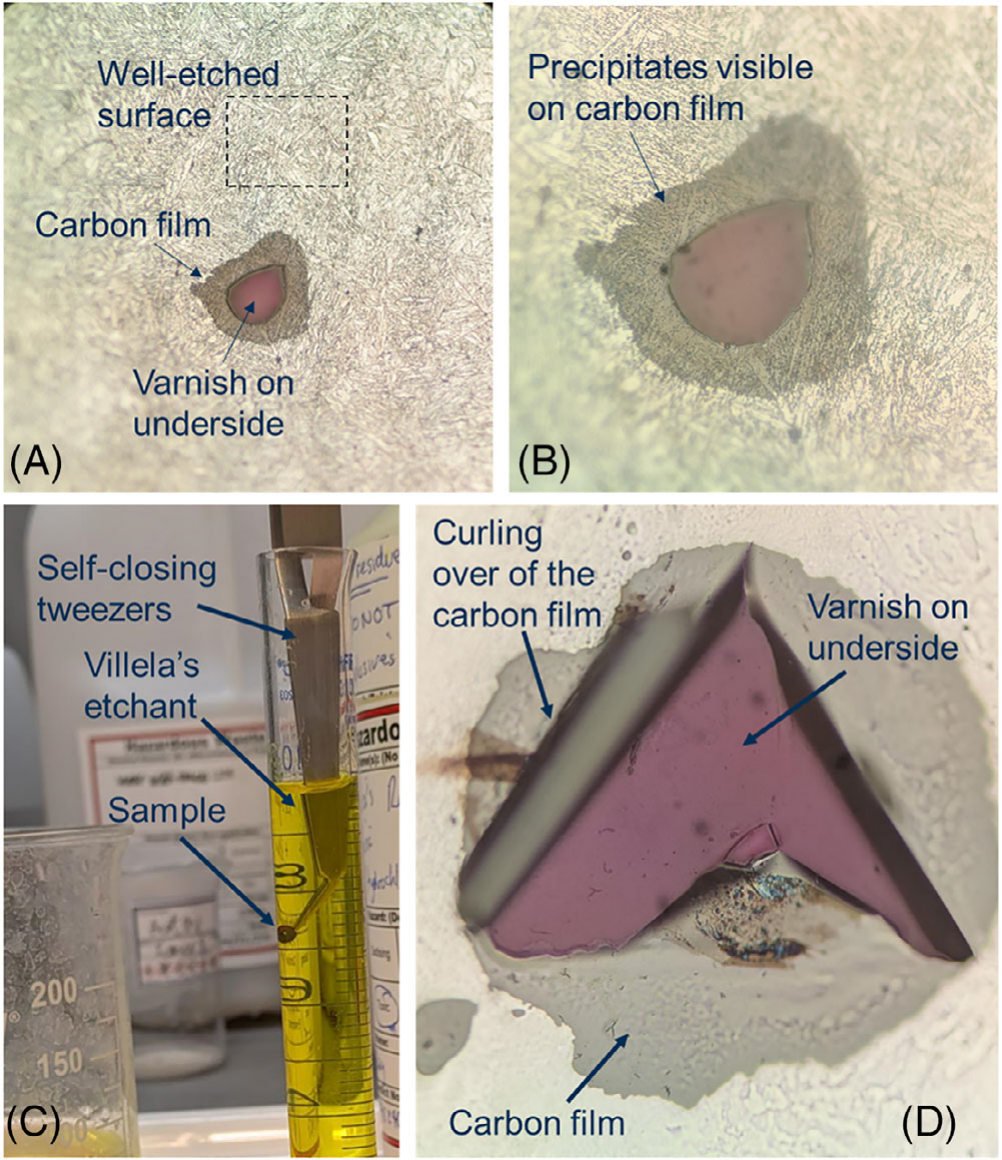
Optical images of different stages of preparation. (A, B) The electropolished hole in a 3 mm disk after the carbon‐coating and etching. Visible are the etched metal surface, a dark‐grey ring around the hole corresponding to the carbon film, and a pink interior showing the protective varnish on the underside. (C) The etching step in practice, where the sample is held by self‐closing tweezers and submerged in Villela's etchant. (D) An example from a different sample (an oxide dispersion strengthened steel), where the carbon film has torn and started to curl up over itself.

Figure 2 shows a series of images from the preparation process, including optical micrographs of the typical etch achieved during electropolishing, and closer examination of the carbon film. In some cases, the carbon film can tear and begin to roll up at the edges, as shown in Figure 2D. This could be avoided if the twin‐jet electropolishing is stopped just prior to perforation, though this is difficult to achieve reliably; the Bergqvist method may be easier if this is problematic.

The Lacomit varnish visible on the underside of the sample in Figure 2A, B, and D is an alcohol‑based, fast‑drying protective lacquer commonly used in metallographic preparation. It forms a chemically resistant coating that withstands etching and electropolishing solutions, allowing selected regions of the specimen to remain unaltered during thinning.

Dissolution of the thinned metal was carried out using Villela's etchant, as shown in Figure 2C. Villela's is a relatively mild etchant for Fe‑based alloys, providing a slow and controllable dissolution rate that avoids undercutting the carbon film or dislodging precipitates. This controlled behaviour is important for the extraction‑replica preparation, as it allows the exposed carbon area around the perforation to be enlarged gradually while preserving the integrity of the replica. The dissolution rate can be adjusted by varying the etchant concentration, and the progress monitored periodically under an optical microscope during the ∼5‐min immersion.

For more corrosion‑resistant alloys, which dissolve very slowly or not at all in Villela's, a stronger or alternative etchant may be required, or flash electropolishing can be used instead. This allows the dissolution step to be tailored to the alloy's specific etching or electropolishing behaviour, ensuring reliable removal of the remaining matrix without damaging the carbon film or precipitates.

### 2.2 Material

An example of this extraction replica technique being used to analyse precipitates in reduced‐activation ferritic martensitic (RAFM) steel is presented here. UKAEA are currently embarking on a rapid alloy development programme called ‘NEURONE’,^12^ with an aim to produce a new RAFM steel targeting higher temperatures than current conventional RAFM alloys, which are typically limited to 550°C. As part of the development, UKAEA are evaluating the capability and readiness of UK steel industry for delivering tonnage‐scale fusion‐grade steels. This is an important endeavour to ensure that any new steels developed are scalable and also that the UK steel industry is primed for the potential surge in demand of high‐quality fusion‐grade steels for the next generation of large‐scale fusion devices. Historically, UK involvement in the development of conventional RAFM steel has been limited to a small lab‐scale batch of Eurofer‐97 produced by British Steel.^13^

As part of the NEURONE programme, a tonnage‐scale heat of a RAFM steel was produced at the Materials Processing Institute, Middlesbrough, intended as a precursor to the scale‐up (from 10s of kg) of the most promising new compositions being developed by UKAEA and their partners.^14^ The heat was produced via Electric‐Arc Furnace (EAF) and aimed to replicate Eurofer‐97 chemistry (see Table 1). A 5.5 tonne billet, dubbed ‘UK‐RAFM’ was produced via continuous casting, which was subsequently forged and rolled, with final normalising and temper treatments consistent with Eurofer‐97 specification (980°C normalisation and 740°C temper). The alloy is undergoing evaluation to assess whether the resulting material is consistent with mechanical and microstructural properties reported in literature for Fusion‐for‐Energy^15^ Eurofer‐97 heats. Among the items requiring characterisation are the SPPs, which must be compared against those characterised in the literature. For this, a study of SPPs in Eurofer‐97 by Klimenkov et al.^16^ is used to benchmark SPP sizes and compositions of the UK‐RAFM heat, specifically the M_23_C_6_ and MX phases. The compositions of the M_23_C_6_ precipitates are of particular interest as they comprise the highest volume fraction of SPPs, and their composition affects their thermal stability.

**TABLE 1:** Measured composition of UK‐RAFM steel, in wt.%.

Fe	C	N	Cr	W	Mn	Si	V	Ta
Bal.	0.11	0.023	10.1	1.0	0.46	0.15	0.22	0.09

This example demonstrates some of the advantages of this new approach to extraction replicas, while also highlighting some of the nuance in quantitative EDS analysis that arises.

### 2.3 Sample preparation

**FIGURE 3:**
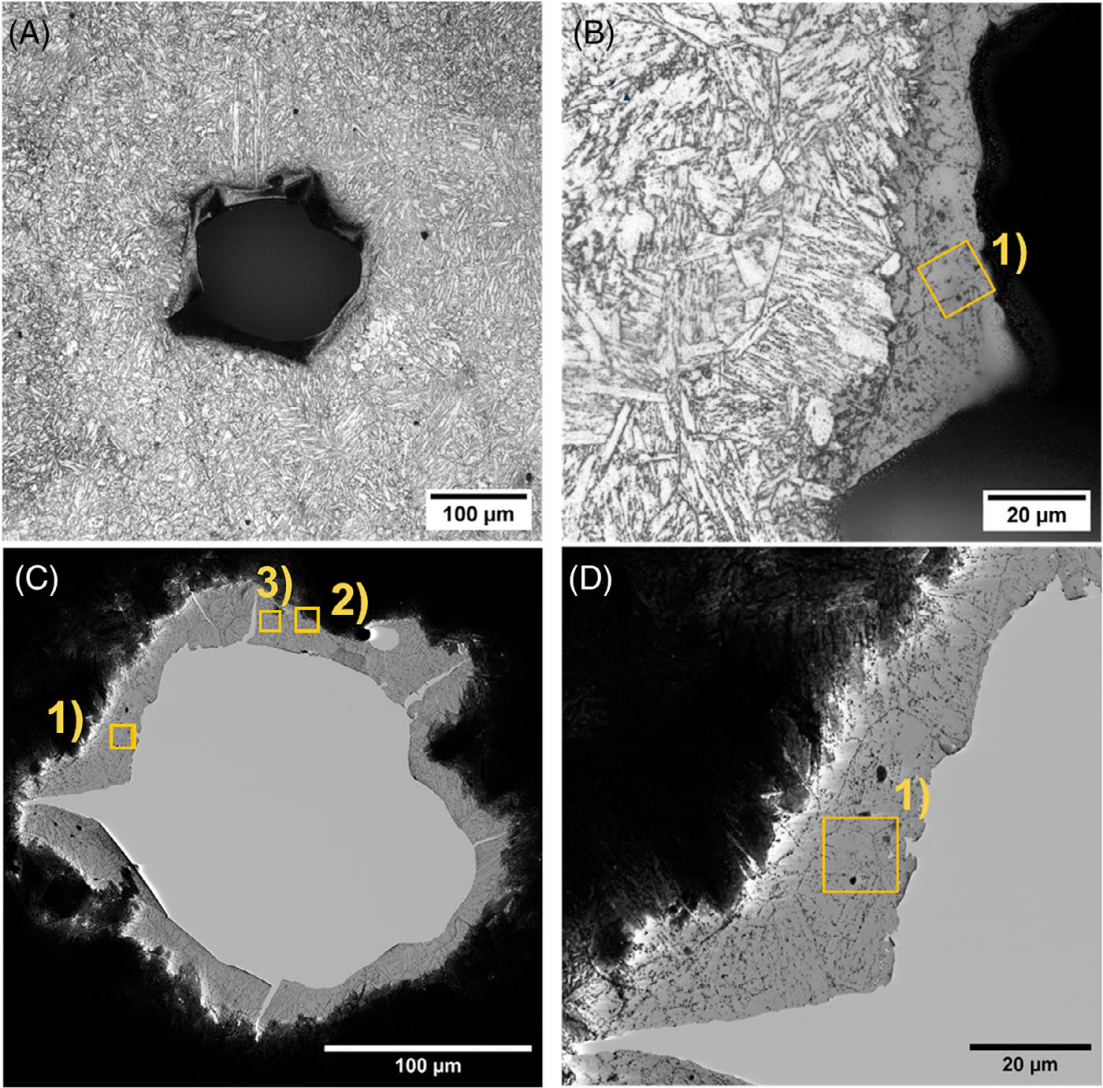
Micrographs showing at low‐magnification the carbon film around the electropolish‐perforated hole of the 3 mm disk. (A, B) Optical images, where the etched martensitic structure can be easily seen. (C, D) Low‐magnification STEM images of the same features images in A and B. The square yellow boxes overlaid and labelled 1 and 2 outline the approximate same areas that were subsequently mapped via STEM‐EDS. Diffraction data was acquired from region 3.

The UK‐RAFM steel was prepared as 3 mm disks, ∼100 µm in thickness, and finished with P1200 grit SiC paper finish. Disks were twin‐jet electropolished at the University of Oxford using a Struers Tenupol‐5 using an electrolyte of 10% perchloric acid (70% concentration), 15% 2‐butoxyethanol, and 75% methanol, and at a temperature between –40°C and –50°C, temperature controlled via periodic addition of liquid nitrogen. Identifying the ideal electropolishing conditions can often be found by plotting applied voltage against current, and observing a plateau in the I‐V curve between etching and pitting conditions that corresponds to even polishing, as depicted in step 2 of Figure 1. However, perchloric solutions do not demonstrate this behaviour.^17^ The best approach is therefore to increase the voltage gradually, and periodically check the surface for signs of etching or pitting. The ideal condition to use for even electropolishing is typically the highest voltage that does not lead to any pitting on the sample.^17^ The optimum voltage can vary across different Tenupol devices; for this particular RAFM steel, we found good electropolishing on our Tenupol‐5 at ∼23 V. With this condition identified, the voltage was then reduced to 13 V to shift the electropolishing condition into etching behaviour. Ideal conditions for the replication process should produce a light etch that reveals a fine distribution of precipitates under optical microscopy, as shown, for example, in Figures 2A and B and 3A and B.

The new method already described for producing the carbon‐extraction replica from the twin‐jet electropolished disk was then applied.

### 2.4 TEM

TEM was performed at the Materials Research Facility (MRF) at UKAEA, on a JEOL NeoARM 200 kV microscope, equipped with cold‐FEG source. EDS maps were acquired using a STEM probe with 70 µm condenser lens aperture, and probe size 1, and JEOL dual‐EDS detectors. Data was analysed using Gatan Microscopy Suite (GMS), Hyperspy,^18^ and ImageJ.^19^, ^20^ Quantitative EDS signals were deconvoluted via GMS, using the Cliff–Lorimer method, with Hyperspy used for further analysis and plotting.

## 3 RESULTS AND DISCUSSION

### 3.1 Microstructural overview

Optical micrographs of the etched surface and the small hole in the centre of the perforated TEM disk are shown in Figure 3. The retained carbon film is visible under optical imaging and compared with STEM.

**FIGURE 4:**
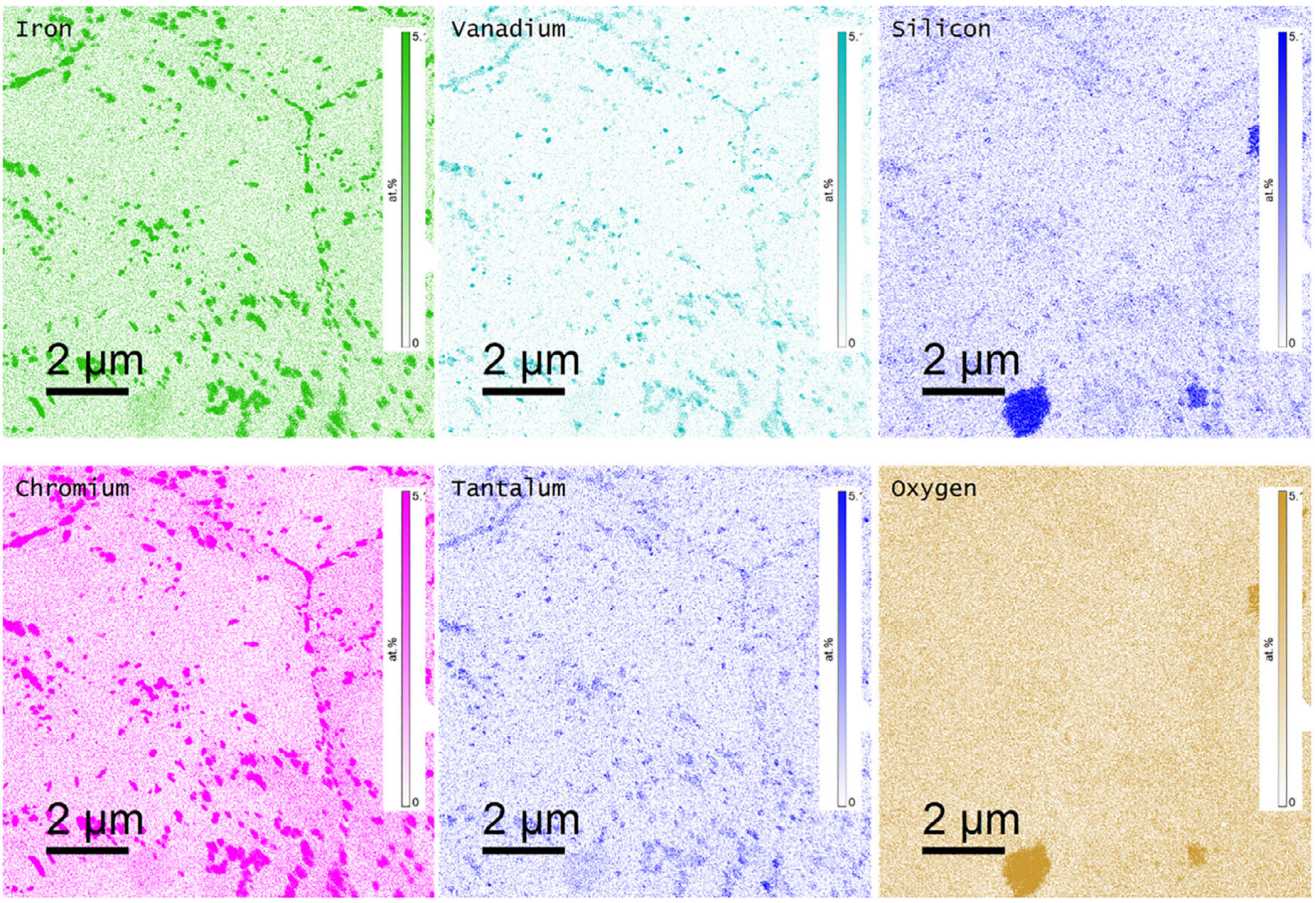
EDS maps around a prior‐austenite boundary triple point (region 1 of Figure 3). The maps were processed using the standard quantification option within GMS, but the contrast has been scaled to aid visualisation. As such, these maps are considered more qualitative. The large defect visible in silicon and oxygen maps is also rich in chlorine (not shown) and is likely an artefact of the electropolishing.

Low‐magnification STEM was used to aid navigation of the sample in the microscope, and correlate the regions observed with the optical images. Figure 3C and D shows a section of the replica from which analytical STEM‐EDS maps were acquired to inspect the precipitate types present and identify the nature of the larger inclusions present here. Figure 4 shows the corresponding STEM‐EDS maps region 1. Note that some defects that appear similar to inclusions are actually rich in chlorine and oxygen, and so are likely to be artefacts from electropolishing with the perchloric electrolytes.

**FIGURE 5:**
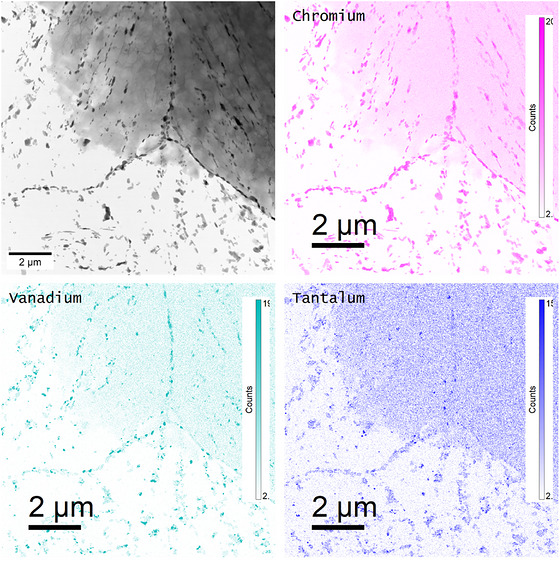
EDS maps around a prior‐austenite boundary triple point (region 2 of Figure 3), overlapping with the metal‐matrix. The maps were processed using the standard quantification option within GMS, but the contrast has been scaled to aid visualisation. As such, these maps are qualitative.

A second area (region 2 in Figure 3), containing both carbon film and metal matrix, is shown in Figure 5 and was used for more detailed analysis. Within the EDS maps (particularly the V and Ta maps), a larger number of small (<50 nm) precipitates are visible in the carbon‑film region than in the metal‑matrix region. In contrast, STEM‑bright‑field (BF) imaging reveals more small precipitates in the metal matrix than on the carbon film. We attribute these BF‑visible precipitates to TaC, as V(C,N) typically exhibits weak diffraction contrast in BF imaging.

This behaviour reflects a well‑known limitation of extraction‑replica preparation: small precipitates are collected less efficiently on the carbon film than larger ones. As a result, the population of precipitates observed in a carbon extraction replica is inherently biased toward larger precipitates.^21^ However, the precipitates that *are* collected on the carbon film provide significantly better EDS signal‑to‑noise than those embedded in the matrix, because there is no surrounding matrix to obscure or dilute the precipitate signal. Consequently, fewer total counts are required to detect a given element in precipitates on the carbon film compared with precipitates within the matrix.

**FIGURE 6:**
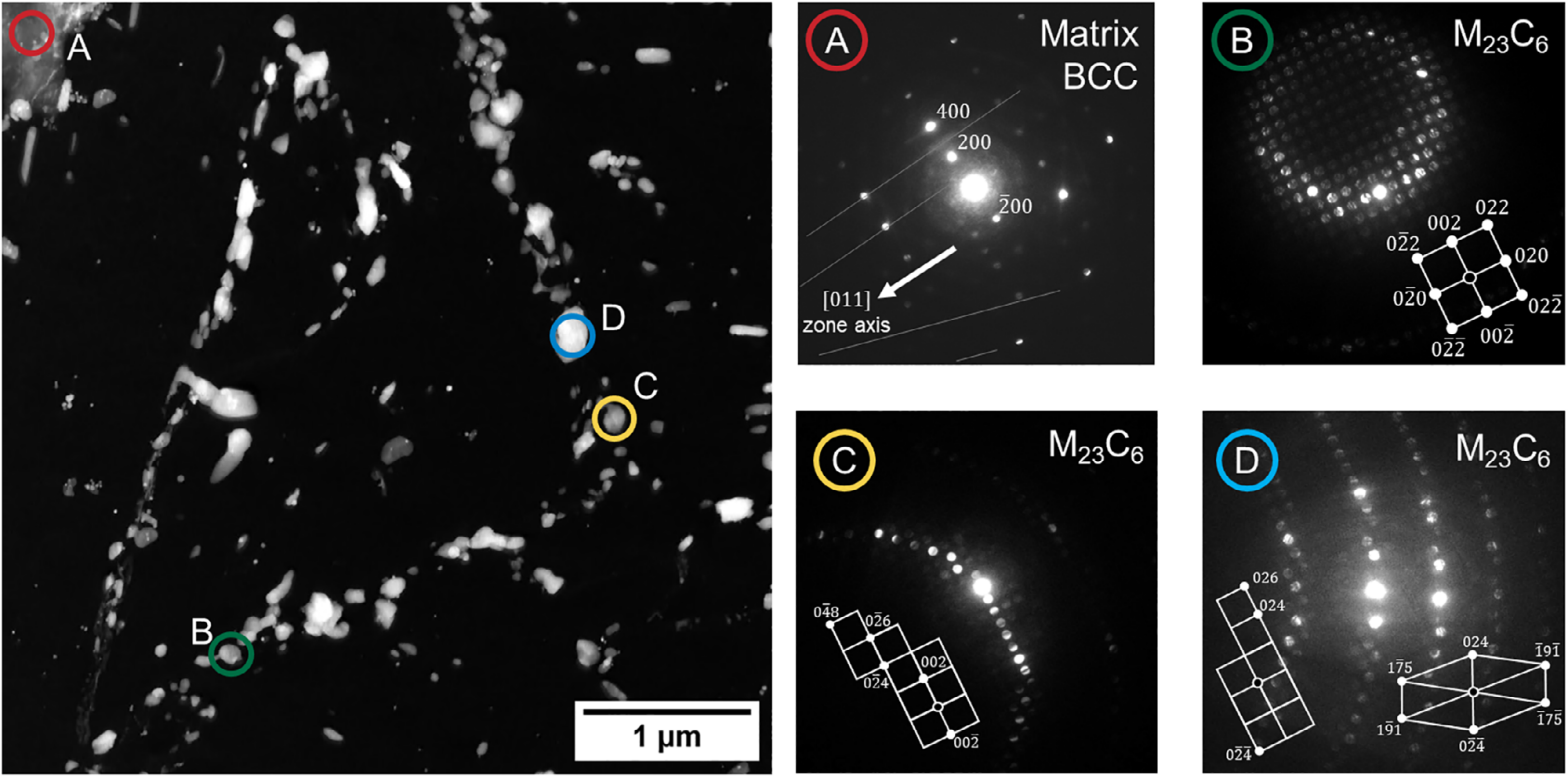
Diffraction patterns acquired from several precipitates in the replica. Region A is part of the matrix, shown to be close to a [011] zone axis, with strong 200 reflections. This was used to calibrate the scale of the patterns. Regions B, C, and D were indexed as M_23_C_6_, with a lattice parameter of 10.5 Å, with respect to an iron lattice parameter of 2.87 Å.

Without matrix to interfere with the precipitate, diffraction patterns from precipitates can also be used to validate their structure, which is otherwise inferred from their composition. Figure 6 shows a diffraction pattern (captured using a pencil‐beam STEM probe) from several large carbides (captured from region 2 in Figure 3). EDS spectra for each confirmed chromium contents of 64–69 at%, which supports our interpretation of the chromium‐rich precipitates being M_23_C_6_. Mapping the diffraction data (i.e. 4D‐STEM) can also be used to offer finer spatial resolution of the microstructure, however this was not done here.

### 3.2 Processing of precipitate EDS data

**FIGURE 7:**
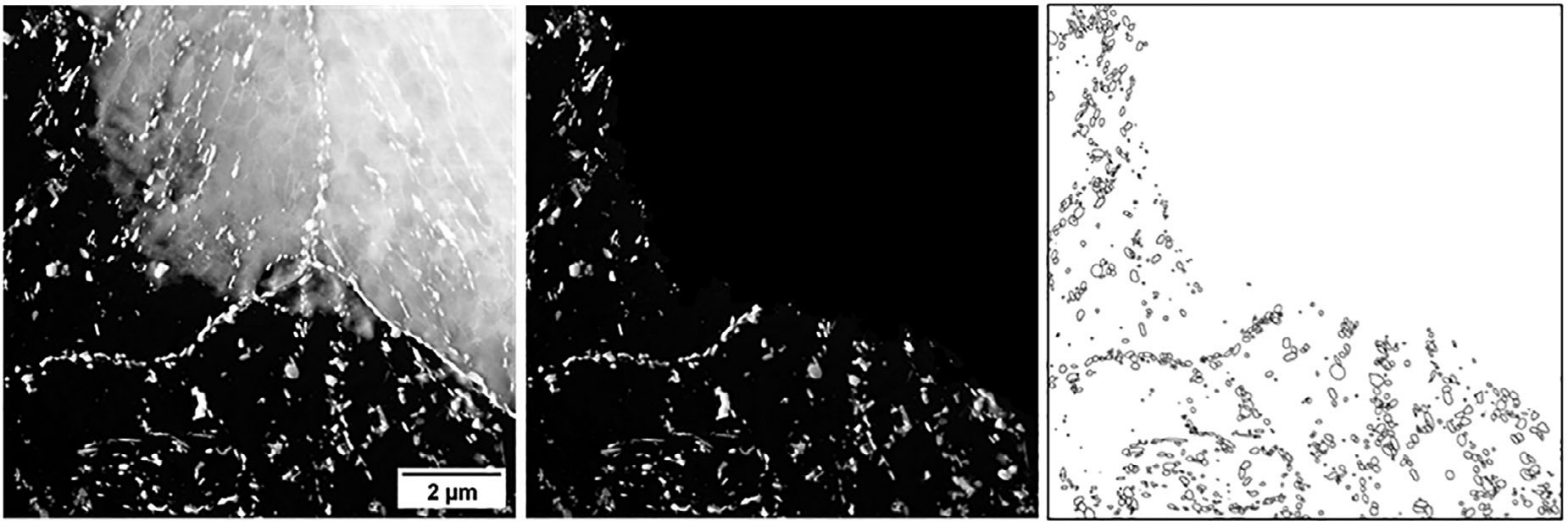
Segmentation of the ADF image to isolate individual precipitates, with the metal‐matrix excluded.

To provide a more robust quantitative analysis of the carbides, the example shown in Figure 5 was segmented using Weka Segmentation^20^ applied to the annular dark‑field (ADF) image. The resulting classification was then refined in ImageJ^19^ using the ROI Manager to define regions of interest (ROIs) for each carbide (Figure 7). This analysis was restricted to carbides located on the carbon film to minimise any influence from the surrounding matrix on the chemical measurements. The EDS map was acquired with a step size of ∼10 nm, meaning that precipitates smaller than ∼20 nm are not captured in the subsequent analysis.

On a per‑pixel basis, the X‑ray counts in these maps are low, so quantitative analysis at the acquired spatial resolution carries substantial uncertainty. However, once the carbides are segmented using the ADF signal, the total counts from all pixels within each carbide can be summed, providing significantly improved statistics for EDS quantification. Only the metallic elements were quantified. Light‑element quantification (particularly carbon) was avoided because: (i) the energy resolution of EDS leads to high uncertainty; (ii) the C‑Kα peak overlaps with several elements present in the alloy; and (iii) the carbides lie on a carbon support film, making it impossible to reliably separate film carbon from carbide carbon.

A limitation of extraction replicas prepared from electropolished disks is the persistent background signal originating from the bulk alloy, as the metal of the 3 mm disc remains present beneath the carbon film. This background must be removed prior to quantification. In addition, the bremsstrahlung continuum is higher over precipitates than over the carbon film due to their greater mass‑thickness, and this correlates with an increased number of stray background peaks. Copper provides a useful indicator of this effect: it is absent from the alloy but present in the background signal due to the copper washer and grub screw in the TEM holder.

**FIGURE 8:**
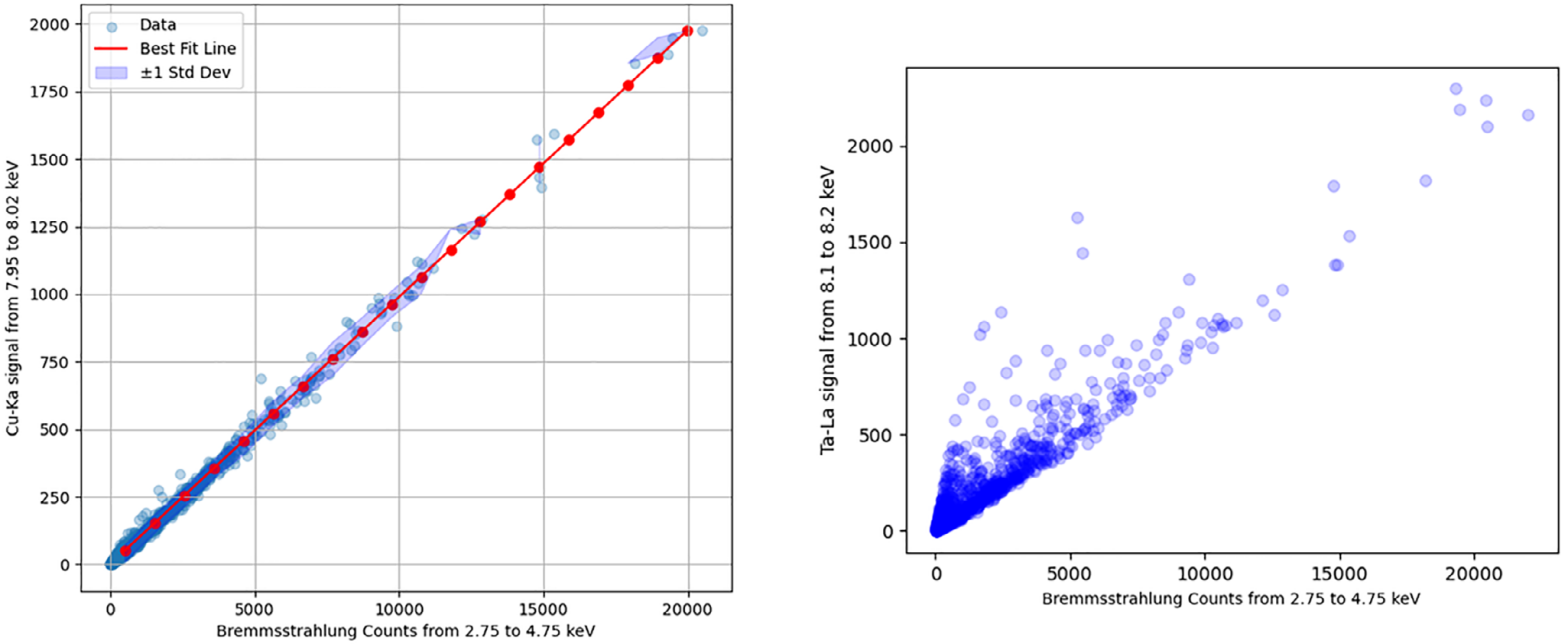
Counts from Cu‐Kα in each precipitate as a function of the bremsstrahlung counts in the range 2.25–4.00 keV. The linear fit has a gradient of 0.128. This is compared to the same plot for tantalum, which comprises convoluted counts from TaC, counts from background Ta in the foil, and overlapped Cu‐Kα counts.

To illustrate this behaviour, Figure 8 plots the Cu‑Kα signal against the total counts in the 2.75–4.75 keV window, which contains no characteristic peaks. For this demonstration, Cu‑Kα was integrated from 7.95 to 8.02 keV, slightly below the nominal 8.04 keV peak, to minimise overlap with Ta‑Lα. The same comparison was made for Ta‑Lα, integrated from 8.1 to 8.2 keV (peak at 8.15 keV). The bremsstrahlung signal increases linearly with Cu‑Kα, whereas the Ta‑Lα plot shows two populations: one following the same linear trend as Cu‑Kα, and a second, shallower trend attributed to regions richer in Ta, which also increases bremsstrahlung counts. These observations demonstrate that the background copper signal scales with the bremsstrahlung continuum. We therefore assume that other background‑derived peaks, particularly those expected from the steel, scale in the same manner.

To remove this background, a spectrum was averaged from a large area of carbon film free of visible precipitates. The continuum level was estimated from the total counts in the 2.75–4.75 keV window. For each precipitate, this background spectrum was normalised so that its counts in this window matched those in the precipitate spectrum. The normalised background spectrum therefore approximates the specific background contribution for that precipitate and can be subtracted accordingly. This approach also removes the copper signal, which would otherwise overlap strongly with Ta‑Lα.

### 3.3 Quantitative analysis of precipitates

With the background removed spectra, GMS was used to deconvolute the signals associated with the vanadium, chromium, manganese and iron K‐series peaks, and the tantalum and tungsten L‐series peaks. Cliff–Lorimer factors were used to weight the signals. The compositions are reported in Tables 2, 3, 4, with each precipitate type grouped after inspection of the overall composition distribution.

**TABLE 2:** Composition analysis and comparison for M_23_C_6_ and average size (equivalent circle diameter).

Precipitate type	M_23_C_6_ (Cr>40%)	M_23_C_6_ (Ta →0)	M_23_C_6_ Thermo‐Calc UK‐RAFM	M_23_C_6_ Klimenkov et al. Eurofer‐97 (83699 plate)	M_23_C_6_ Thermo‐Calc Eurofer‐97 (83699 plate)
Cr	69.4 (62.3–72.4) {2.0}	69.7 (62.7–72.7) {2.0}	67	70 ± 1	66
W	2.4 (1.5–3.5) {0.3}	2.4 (1.5–3.5) {0.3}	5.4	2.4 ± 0.2	6.0
V	0.9 (0.24–2.5) {0.2}	0.9 (0.2–2.7) {0.2}	2.7	1.1 ± 0.2	2.5
Fe	26.9 (21.3–30.7) {1.0}	26.9 (21.2–30.8) {1.0}	24	27 ± 1	26
Ta	0.4 (0.1–0.8) {0.1}	–	0.0	–	
Mn	0.0 (0.0–0.5) {0.1}	0.0 (0.0–0.5) {0.1}	1.0	–	1.1
Fe:Cr ratio	0.39 (0.29–0.48) {0.02}		0.36	0.39	0.39
ECD (nm)	105 (79–136)	105 (79–136)		110 ± 4	
Weighted ECD (nm)	86 (69–116)	86 (68–115)			

**TABLE 3:** Composition analysis for V(C,N) and average size (equivalent circle diameter).

Precipitate type	V(C,N) (V>50%)	V(C,N) (Fe, Mn, W → 0)	VN Thermo‐Calc UK‐RAFM	V(C,N) Klimenkov et al. Eurofer‐97 (83699 plate)	VN Thermo‐Calc Eurofer‐97
Cr	6.6 (0.0–13.9) {1.7}	4.6 (0.0–13.0) {1.6}	1.8	4.9 ± 0.5	2.0
W	0.0 (0.0–0.0) {0}	–			
V	87.0 (71.4–88.4) {8.3}	89.3 (74.0–92.2) {8.2}	95	91 ± 2.8	94
Fe	0.0 (0.0–1.2) {0.7}	–	0.2		0.2
Ta	6.4 (3.4–10.1) {2.0}	6.1 (3.1–9.4) {2.0}	3.3	4.6 ± 0.6	3.8
Mn	0.0 (0.0–0.2) {0.2}	–			
ECD (nm)	54 (40–67)	54 (40–67)		48 ± 4	
Weighted ECD (nm)	44 (32–58)	44 (32–58)			

**TABLE 4:** Composition analysis for TaC and average size (equivalent circle diameter).

Precipitate type	TaC (Ta>50%)	TaC (Fe, Mn, W → 0)	TaC Thermo‐Calc UK‐RAFM	TaC Klimenkov et al. Eurofer‐97 (83699 plate)	TaC Thermo‐Calc Eurofer‐97
Cr	0.0 (0.0–3.0) {2.4}	0 (0–0) {0}	5.7		5.6
W	0.0 (0.0–0.0) {0.0}	–	0.1		0.1
V	8.1 (1.3–21.7) {5.5}	7.0 (1.3–22.4) {5.7}	5.2	31 ± 4.2	5.6
Fe	0.2 (0.0–3.3) {2.7}	–			
Ta	91.6 (70.1–91.3) {19.0}	93.0 (76.3–97.4) {19.0}	89	70 ± 1.2	89
Mn	0.0 (0.0–1.2) {1.4}	–			
ECD (nm)	43 (36–53)	43 (36–53)		45 ± 2	
Weighted ECD (nm)	40 (32–49)	40 (34–49)			

**FIGURE 9:**
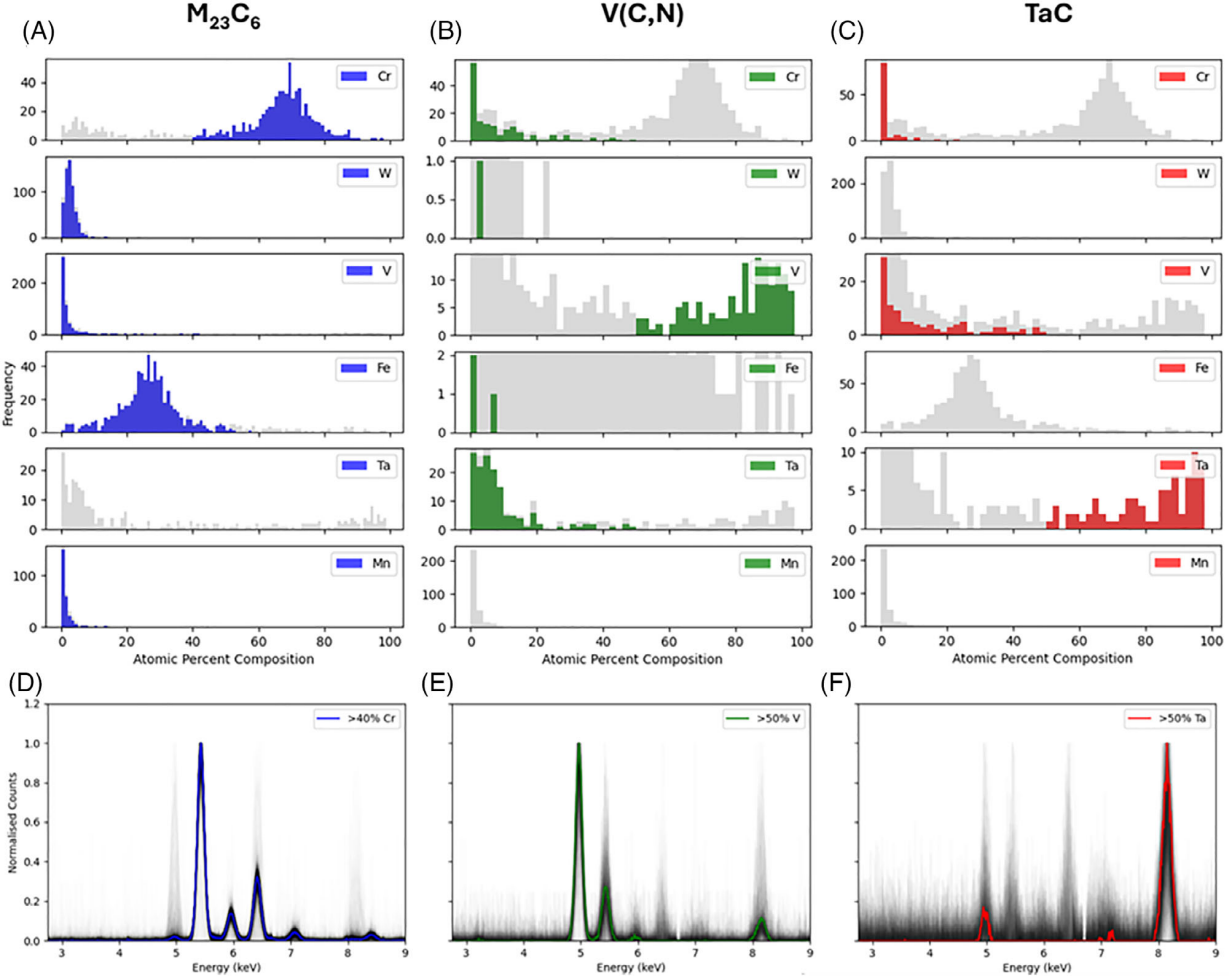
Composition distribution plots for the grouped precipitates, designated M_23_C_6_ (A), V(C,N) (B), and TaC (C). Grey parts of each represent the total composition distribution. Also shown (D–F) are the average spectrum from each of these groups, overlaid atop the individual spectra of all precipitates in the group. Lines of note are V‐Kα (4.95 keV), Cr‐Kα (5.41 keV), Mn‐Kα (5.90 keV), Fe‐Kα (6.40 keV), Fe‐Kβ (7.06 keV), Ta‐Ll (7.17 keV), Ta‐Lα (8.15 keV) and W‐Lα (8.40 keV).

For M_23_C_6_, the M component for these steels is expected to comprise mostly chromium and iron. From inspection of the composition distribution (Figure 9), assignment of M_23_C_6_ as those >40% Cr was considered a reasonable threshold. For MX, TaC and V(C,N) are expected, however other elements can also be present in smaller fractions such as chromium, or tantalum and vanadium in the same MX precipitate. A reasonable threshold to group the MX precipitates was therefore assigned for V(C,N) as those >50% V, and for TaC as those >50% Ta.

Tables 2, 3, 4 show the median composition of the M_23_C_6_, V(C,N), and TaC precipitate groups, with the interquartile range shown in brackets. Uncertainty for each element was estimated from Poisson counting statistics, using $\sqrt{N}$ for the net counts after background subtractions. These uncertainties were then propagated through the Cliff–Lorimer quantification for each precipitate. The 75th percentile of the resulting composition errors is shown in curly brackets in Tables 2, 3, 4 and is taken as a conservative estimate of the overall composition uncertainty. Precipitate sizes are reported as equivalent circle diameters (ECD).

For all elements in M_23_C_6_, there is a wide interquartile range that exceeds the composition uncertainty. This seems to suggest that the observed variation in composition cannot be attributed to measurement uncertainty alone. Additional scatter is to be expected from the close proximity of V(C,N) to many M_23_C_6_, which will contribute a reduction in the measured chromium content, and thus scatter in the distribution below the average of ∼70%.

The composition distributions for each of the precipitate groups are shown in Figure 9A–C, and average spectrum for the group also shown in Figure 9D–F. Inspection of these histograms confirms the metal content of M_23_C_6_ is dominated by chromium and iron. Vanadium is also present, though this is difficult to quantify accurately due its expected presence in both the M_23_C_6_ phase and V(C,N), which can be in close proximity. V(C,N) are, by definition, dominated by vanadium, but there are peaks in the composition distribution for chromium and tantalum, implying that there is a preferred stoichiometry has been captured. However, a large fraction of these also had zero chromium or zero tantalum.

Compared to the Thermo‐Calc^22^, ^23^ prediction (also reported in Tables 2, 3, 4), the measured composition of M_23_C_6_ is mostly similar, but measured tungsten is much lower (5.2 at.% predicted, 2.4 at.% measured). Klimenkov et al.^16^ report a similar composition also for Eurofer‐97 (83699), though manganese was omitted due to low signal, and the tungsten was also slightly higher. Klimenkov et al. tungsten measurements were much less than that predicted by Thermo‐Calc, matching the finding from UK‐RAFM in Table 2. Klimenkov et al. also analysed aged samples, however, and observed increasing chromium in the M_23_C_6_, decreasing iron, but with tungsten changing only slightly. This suggests that Thermo‐Calc is overpredicting the tungsten in these carbides, rather than the alloys being too far from a thermal‐equilibrium condition.

There is also disparity between the composition measured for V(C,N) compared against Thermo‐Calc; chromium as measured here and by Klimenkov et al. is higher than Thermo‐Calc predicts. There is more scatter in the TaC data, owing to their lower frequency in this dataset. Chromium is absent from most of the TaC measured here, however, whereas ∼5at% is predicted by Thermo‐Calc.

**FIGURE 10:**
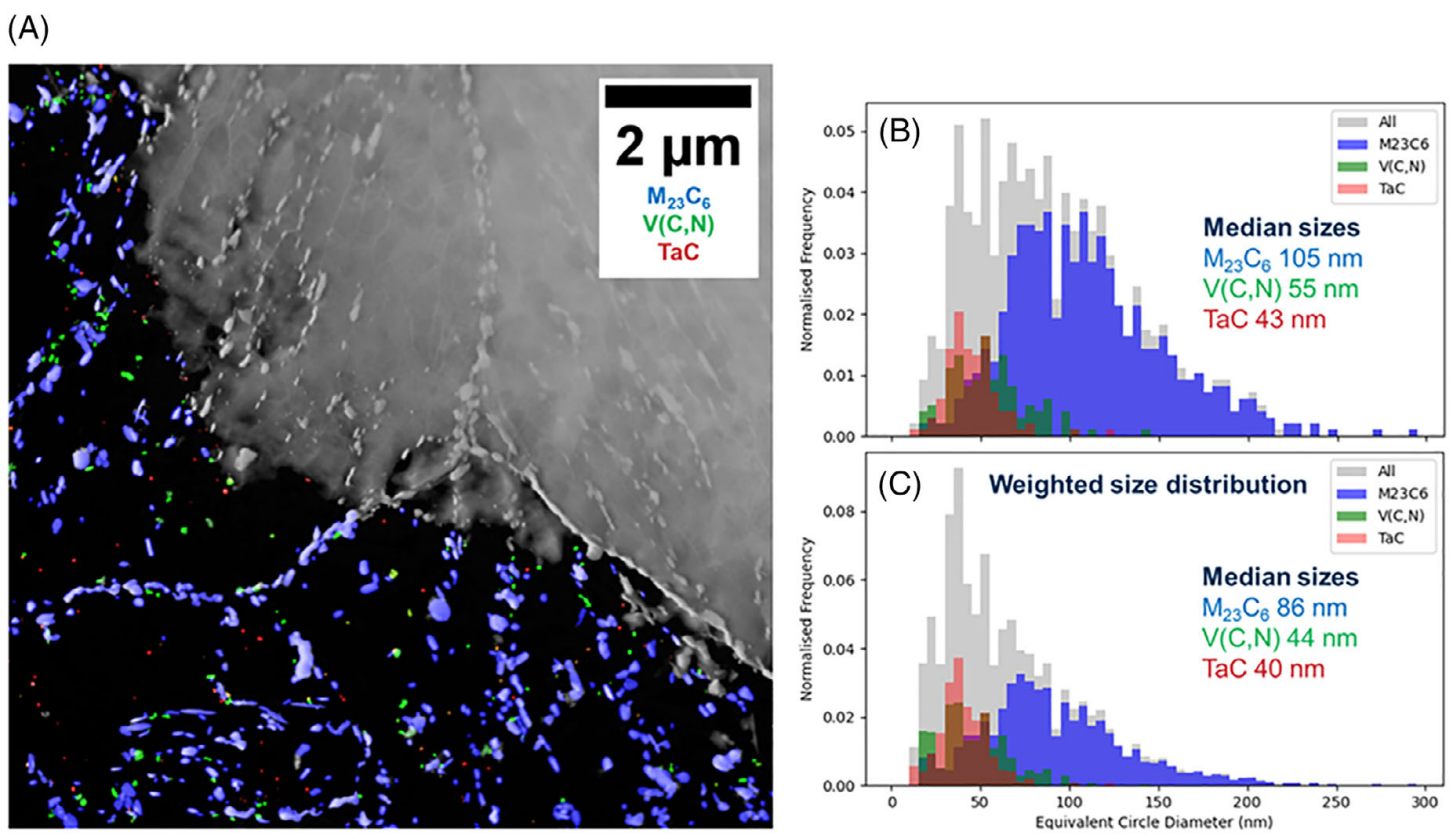
(A) ADF image of the EDS‐mapped region of the UK‐RAFM, with precipitates coloured according to type. (B) The normalised size distribution of the precipitates as recorded, and (C) as weighted according to size, to account for the size‐bias in extraction replica samples.

The sizes given for the precipitates in the UK‐RAFM alloy are also similar to those reported in Eurofer‐97 by Klimenkov et al. In Figure 10, the ADF image is overlaid with the segmented precipitates, now coloured according to the three precipitate groups used throughout this manuscript. Alongside this are two histograms showing the sizes of the precipitates, taken as the equivalent circle diameter (ECD). One shows the size distribution as recorded, with no correction made to account for the distribution for extraction replicas being skewed towards larger sizes. These sizes are more comparable to the measurements made by Klimenkov. A more representative size distribution is shown as the weighted size distribution, to account for each bin of the histogram containing a precipitate population (with average size D), having been extracted from a volume V, where V ∝ D. This weights the precipitates according to 1/D. This problem with extraction replicas is discussed by Ashby and Ebeling.^21^

## 4 CONCLUSIONS

A novel approach to producing carbon‐extraction replicas has been demonstrated via carbon‐coating and etching twin‐jet electropolished TEM disks. By avoiding the need to float‐off the carbon film, the location of the extracted precipitates with respect to the bulk sample is preserved. We have provided an example of this sample preparation being used to analyse the composition of precipitates in an RAFM steel and made comparisons to reported literature.

## Data Availability

The data that support the findings of this study are available from the corresponding author upon reasonable request.
